# Evaluation of Different Versions of the Duke Criteria for the Diagnosis of Infective Endocarditis Among Patients With Enterococcal Bacteremia; a Multicenter Study

**DOI:** 10.1093/ofid/ofaf408

**Published:** 2025-07-04

**Authors:** Virgile Zimmermann, Nicolas Fourré, Bruno Ledergerber, Jana Epprecht, Berit Siedentop, Pierre Monney, Georgios Tzimas, Michelle Frank, Laurence Senn, Nicoleta Ianculescu, Lars Niclauss, Matthias Kirsch, Mathias Van Hemelrijck, Omer Dzemali, Benoit Guery, Barbara Hasse, Matthaios Papadimitriou-Olivgeris

**Affiliations:** Infectious Diseases Service, Lausanne University Hospital and University of Lausanne, Lausanne, Switzerland; Infectious Diseases Service, Lausanne University Hospital and University of Lausanne, Lausanne, Switzerland; Department of Infectious Diseases and Hospital Epidemiology, University Hospital Zurich and University of Zurich, Zurich, Switzerland; Department of Infectious Diseases and Hospital Epidemiology, University Hospital Zurich and University of Zurich, Zurich, Switzerland; Department of Infectious Diseases and Hospital Epidemiology, University Hospital Zurich and University of Zurich, Zurich, Switzerland; Department of Cardiology, Lausanne University Hospital and University of Lausanne, Lausanne, Switzerland; Department of Cardiology, Lausanne University Hospital and University of Lausanne, Lausanne, Switzerland; Department of Cardiology, University Hospital Zurich and University of Zurich, Zurich, Switzerland; Infectious Diseases Service, Lausanne University Hospital and University of Lausanne, Lausanne, Switzerland; Infection Prevention and Control Unit, Lausanne University Hospital and University of Lausanne, Lausanne, Switzerland; Department of Cardiology, Lausanne University Hospital and University of Lausanne, Lausanne, Switzerland; Department of Cardiac Surgery, Lausanne University Hospital and University of Lausanne, Lausanne, Switzerland; Department of Cardiac Surgery, Lausanne University Hospital and University of Lausanne, Lausanne, Switzerland; Department of Cardiac Surgery, University Hospital Zurich and University of Zurich, Zurich, Switzerland; Department of Cardiac Surgery, University Hospital Zurich and University of Zurich, Zurich, Switzerland; Infectious Diseases Service, Lausanne University Hospital and University of Lausanne, Lausanne, Switzerland; Department of Infectious Diseases and Hospital Epidemiology, University Hospital Zurich and University of Zurich, Zurich, Switzerland; Infectious Diseases Service, Lausanne University Hospital and University of Lausanne, Lausanne, Switzerland; Infectious Diseases Service, Hospital of Valais and Institut Central des Hôpitaux, Sion, Switzerland

**Keywords:** bloodstream infection, Duke criteria, enterococci, *Enterococcus faecalis*, infective endocarditis

## Abstract

**Background:**

Enterococci are a common cause of infective endocarditis (IE). This study aimed to assess the diagnostic performance of the 2015 and 2023 Duke versions of the European Society of Cardiology (ESC) Duke criteria, as well as the 2023 Duke International Society of Cardiovascular Infectious Diseases (ISCVID) clinical criteria, for identifying IE among patients with enterococcal bacteremia.

**Methods:**

We included adult retrospective patients with enterococcal bacteremia from 3 independent cohorts across 2 Swiss university hospitals between 2015 and 2024. An interdisciplinary Endocarditis Team classified each case as either IE or not IE. Each episode was then classified as definite, possible, or rejected IE according to the 2015 Duke-ESC, 2023 Duke-ESC, and 2023 Duke-ISCVID clinical criteria. Patients with IE (reference standard) classified as definite IE by the Duke criteria were considered true positives, while those without IE classified as rejected IE were considered true negatives.

**Results:**

Among 827 episodes with enterococcal bacteremia, IE was diagnosed in 173 (21%) episodes. The sensitivity of the 2015 Duke-ESC, 2023 Duke-ISCVID, and 2023 Duke-ESC clinical criteria for diagnosing IE was 67% (95% CI, 59%–74%), 79% (95% CI, 72%–85%), and 74% (95% CI, 67%–80%), respectively. Specificity was 86% (95% CI, 83%–89%) for the 2015 Duke-ESC criteria, 55% (95% CI, 51%–59%) for the 2023 Duke-ISCVID criteria, and 69% (95% CI, 65%–72%) for the 2023 Duke-ESC criteria.

**Conclusions:**

Among the evaluated Duke criteria versions, the 2023 Duke-ISCVID criteria demonstrated the highest sensitivity for diagnosing IE in patients with enterococcal bacteremia. However, this was at the expense of specificity.

Enterococci, particularly *Enterococcus faecalis*, are among the most common causes of infective endocarditis (IE) [1], ranking behind *Staphylococcus aureus* and streptococcal species in frequency [2, 3]. The incidence of enterococcal IE is notably higher among elderly patients and in individuals with transcatheter aortic valve implantation [1, 4].

Since the introduction of the Duke criteria in 1994, enterococci have been recognized as typical IE pathogens, alongside *S. aureus* and selected streptococcal species, provided that the bacteremia was community acquired and had no identifiable primary focus [5, 6]. In 2015, the European Society of Cardiology (ESC) guidelines updated the Duke criteria by adding cardiac computed tomography (CT) for both native and prosthetic valve IE, as well as ^18^F-fluorodeoxyglucose positron emission tomography/CT (^18^F-FDG PET/CT) for diagnosing prosthetic valve IE [7].

Further revisions followed in 2023, when both the ESC and the International Society for Cardiovascular Infectious Diseases (ISCVID) introduced further adaptations of the Duke criteria [8, 9]. These updates included changes to the major microbiology and imaging criteria, minor predisposing conditions, and vascular phenomena. Importantly, both 2023 criteria revised the list of typical IE pathogens by replacing “community-acquired enterococci without a known primary focus” with “*E. faecalis*,” based on prior analyses that focused on *E. faecalis* bacteremia [10, 11]. Additionally, the 2023 Duke-ISCVID criteria classified other enterococcal species as typical IE pathogens if ≥3 blood culture sets were positive [9]. These modifications demonstrated improved sensitivity in various clinical settings compared with the 2015 Duke-ESC criteria [3, 12–19]. However, studies evaluating the revised microbiological criterion for *E. faecalis* have reported an increased number of cases reclassified from “rejected” to “possible” IE, raising concerns about the appropriateness of categorizing *E. faecalis* as a typical IE pathogen [3, 12].

This study aims to assess the diagnostic accuracy of the different versions of the Duke criteria in identifying IE among patients with enterococcal bacteremia.

## METHODS

This retrospective study was conducted at 2 Swiss university hospitals—Lausanne University Hospital (CHUV) and University Hospital Zurich (USZ)—and included patients from 3 distinct cohorts spanning from January 2015 to June 2024. The first cohort consisted of patients from CHUV's retrospective bacteremia database (January 2015 to December 2021). The second was CHUV's prospective cohort of patients with suspected IE enrolled between January 2022 and June 2024; suspicion of IE was defined by the performance of blood cultures and echocardiography specifically to investigate IE. The third cohort originated from USZ's endocarditis cohort, which included patients retrospectively from 2015 to 2017 and prospectively from 2018 onward. The study received approval from the relevant Swiss ethics committees (CER-VD 2021-02516, CER-VD 2017-02137, KEK-2014-0461, BASEC-2017-01140).

Inclusion criteria encompassed adult patients (age ≥18 years) with ≥1 blood culture for *Enterococcus* spp. and no documented refusal to use their clinical data for research purposes. Exclusion criteria consisted of patients with incomplete medical records (specifically patients transferred to external hospitals at the onset of infection without available follow-up information).

Demographic, clinical, imaging, microbiological, surgical, and pathological data were retrieved from electronic health records. In both institutions, infectious diseases (ID) specialists were routinely notified of all positive blood cultures for enterococci [20]. While ID consultation was generally left to the discretion of the consulting physician, it became mandatory when IE was suspected. In such cases, follow-up blood cultures were systematically obtained until clearance of bacteremia was documented. Diagnosis of IE was established by each hospital's dedicated Endocarditis Team, which has served as the diagnostic reference standard since January 2018. For episodes occurring before 2018, 2 senior clinicians from each institution, who had also been members of their respective Endocarditis Teams since 2018 (CHUV: M.P.O., P.M.; USZ: B.H., M.F.), retrospectively reviewed and adjudicated the cases using the same diagnostic framework. At both centers, the Endocarditis Teams consisted of 2 ID specialists, 2 cardiologists, 2 cardiac surgeons, and 1 nuclear medicine specialist. All cases of suspected IE were referred to weekly Endocarditis Team meetings by the responsible treating physician [21]. Each episode was classified as definite, possible, or rejected IE using all 3 versions of the Duke clinical criteria: the 2015 Duke-ESC [7], the 2023 Duke-ISCVID [9], and the 2023 Duke-ESC criteria [8].

The date of first positive blood culture was defined as the onset of infection. A new episode was included if >30 days had passed since completion of antimicrobial treatment for the previous episode. Bacteremia episodes were classified as community acquired, health care associated, or nosocomial according to the criteria established by Friedman et al. [22]. The site of infection was determined by the ID consultant based on a comprehensive evaluation of clinical presentation, imaging, microbiological findings, and surgical reports.

Data analysis was performed using SPSS, version 26.0 (SPSS, Chicago, IL, USA). Categorical variables were compared using the chi-square or Fisher exact test, while continuous variables were analyzed using the Mann-Whitney *U* test. Two analytical approaches were used to evaluate the diagnostic performance of the Duke clinical criteria for diagnosing IE, using the Endocarditis Teams' or expert clinicians' final diagnosis as the reference standard. In the first analysis, patients with IE (according to the reference standard) who were classified as having definite IE by the Duke criteria were considered true positives, whereas those classified as possible or rejected IE were considered false negatives. Conversely, patients without IE (according to the reference standard) who were classified as rejected or possible IE were considered true negatives, while those classified as definite IE were considered false positives. In the second analysis, the only change concerned patients without IE (according to the reference standard) who were categorized as possible IE by the Duke criteria: These were now considered false positives instead of true negatives. Sensitivity, specificity, positive predictive values (PPVs), negative predictive values (NPVs), and overall diagnostic accuracy were calculated with corresponding 95% CIs. All statistical tests were 2-tailed, with a significance threshold set at *P* < .05.

## RESULTS

**Figure 1.**
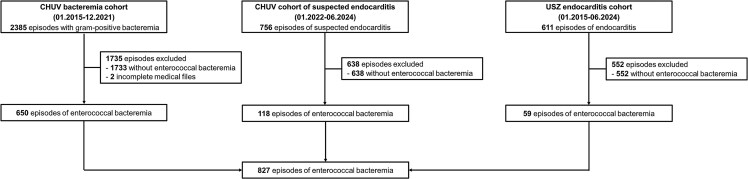
Flowchart of included episodes. Abbreviations: CHUV, Lausanne University Hospital; USZ, University Hospital Zurich.

Out of 3752 episodes included across the 3 cohorts, a total of 828 episodes involving enterococcal bacteremia were analyzed (Figure 1). *E. faecalis* was the most commonly identified species, accounting for 481 episodes (58%), followed by *E. faecium* in 326 (39%) episodes (Table 1). Cardiac imaging was performed in 484 (58%) episodes, including transthoracic echocardiography (TTE) in 442 (53%) episodes, transesophageal echocardiography (TEE) in 175 (21%) episodes, ^18^F-FDG PET/CT in 79 (10%) episodes, and cardiac CT in 13 (2%) episodes. ID consultation was provided in 707 (85%) episodes.

**Table 1.** Comparison of Episodes With or Without Infective Endocarditis Among 828 Episodes With Enterococcal Bacteremia

	No Infective Endocarditis (n = 654)	Infective Endocarditis (n = 173)	*P*
Demographics			
Male sex, No. (%)	455 (70)	128 (74)	.262
Age, median (IQR), y	70 (59–78)	70 (58–79)	.630
Setting of infection onset, No. (%)			
Community acquired	110 (17)	116 (67)	
Health care associated	105 (16)	31 (18)	
Nosocomial	440 (67)	26 (15)	<.001^a^
Cardiac predisposing factors, No. (%)			
Intravenous drug use	9 (1)	21 (12)	<.001
Rheumatic heart disease	1 (0.2)	0 (0)	1.000
Congenital disease	8 (1)	20 (12)	<.001
Prosthetic valve	32 (5)	58 (34)	<.001
Prior endocarditis	14 (2)	28 (16)	<.001
Moderate/severe valve regurgitation/stenosis	34 (5)	22 (19)	<.001
CIED	49 (8)	46 (27)	<.001
Transcatheter aortic valve replacement	5 (0.8)	17 (10)	<.001
Heart transplantation	5 (0.8)	2 (1)	.641
Left ventricular assist device	6 (0.9)	3 (2)	.405
Microbiological data, No. (%)			
≥2 positive blood cultures (initial blood cultures)	356 (54)	154 (89)	<.001
≥3 positive blood cultures (initial blood cultures)	153 (23)	79 (46)	<.001
Species, No. (%)			
*E. faecalis*	327 (50)	154 (89)	<.001
*E. faecium*	308 (47)	18 (10)	<.001
Other enterococcal species	49 (8)	3 (2)	.004
Polymicrobial bloodstream infection	242 (37)	13 (8)	<.001
Persistent enterococcal bacteremia (≥48 h)	59 (9)	32 (19)	.001
Community-acquired enterococci without known primary focus	48 (7)	145 (84)	<.001
Infection data, No. (%)			
Fever	555 (85)	140 (81)	.244
Vascular phenomena (major arterial emboli, septic pulmonary infarcts, mycotic aneurysm, intracranial hemorrhage, conjunctival hemorrhages, and Janeway's lesions)	22 (3)	65 (38)	<.001
Cerebral abscess	1 (0.2)	1 (0.6)	.374
Immunological phenomena	0 (0)	8 (5)	<.001
Bone and joint infection	23 (4)	11 (6)	.128
Septic arthritis	2 (0.3)	6 (4)	.001
Spondylodiscitis	7 (1)	8 (5)	.005
Imaging data, No. (%)			
Positive echocardiography (either TTE or TEE) for vegetation, perforation, dehiscence of prothesis, abscess, aneurysm, pseudoaneurysm, fistula	0 (0)	118 (68)	<.001
Abnormal metabolic activity in ^18^F-FDG PET/CT	0 (0)	22 (13)	<.001
Abnormal metabolic activity in ^18^F-FDG PET/CT in native valve or CIED lead	0 (0)	8 (5)	<.001
Abnormal metabolic activity in ^18^F-FDG PET/CT in prosthetic valve	0 (0)	14 (8)	<.001
Positive cardiac CT for vegetation, perforation, dehiscence of prothesis, abscess, aneurysm, pseudoaneurysm, fistula	0 (0)	8 (5)	<.001
Types of cardiac lesion, No. (%)			
Vegetation	0 (0)	114 (66)	<.001
Abscess	0 (0)	25 (15)	<.001
Perforation, dehiscence of prothesis	0 (0)	19 (11)	<.001
Aneurysm, pseudoaneurysm, fistula	0 (0)	6 (4)	<.001
Leaflet thickening	3 (0.5)	23 (13)	<.001
Significant new valvular regurgitation	16 (2)	60 (35)	<.001
Data on surgery/CIED extraction/histopathology, No. (%)			
Valve surgery performed	0 (0)	62 (36)	<.001
Macroscopic evidence of IE by inspection	0 (0)	40 (23)	<.001
CIED extraction (n = 91 episodes with CIED)	0 (0)	10 (22)	.002
Autopsy performed	6 (0.9)	2 (1)	.675
Duke pathological criterion	0 (0)	31 (18)	<.001

Abbreviations: ^18^F-FDG PET/CT, ^18^F-fluorodeoxyglucose positron emission tomography/computed tomography; CIED, cardiac implantable electronic device; CT, computed tomography; IE, infective endocarditis; IQR, interquartile range; TEE, transesophageal echocardiography; TTE, transthoracic echocardiography.

^a^Comparison between nosocomial bacteremias and both community and health care associated.

In total, 173 (21%) episodes were diagnosed as IE by the Endocarditis Team or expert clinicians. Of these, 100 (58%) episodes involved native valves, 64 (37%) involved prosthetic valves, and 22 (13%) were associated with cardiac implantable electronic device (CIED) leads. Recurrence of bacteremia with the same *Enterococcus* species within 120 days occurred in 7 of 173 (4%) episodes, 6 of which were confirmed as recurrent IE (Supplementary Table 1). Among the 655 episodes not classified as IE, the majority were attributed to abdominal infections (307 episodes; 47%) or catheter-related bloodstream infections (133 episodes; 20%). Recurrent bacteremia with the same *Enterococcus* species occurred in 32 of these non-IE episodes (5%) within 120 days of the initial infection. Of these, 3 were subsequently diagnosed as IE, while the remaining 29 episodes were confirmed as non-IE cases (Supplementary Table 1).

Among the 650 episodes in the CHUV's bacteremia cohort, the overall prevalence of IE was 7% (47 episodes) (Supplementary Table 2). IE was more frequently diagnosed in cases involving *E. faecalis* (41 of 335 episodes; 12%) compared with both *E. faecium* (6 of 295; 2%) and other *Enterococcus* species (1 of 47; 2%).

Of the 828 total episodes, 344 (42%) did not undergo any form of cardiac imaging. Among these, 340 (99%) had a clearly identified alternative infectious focus, and 339 (99%) were treated with antimicrobial treatment for ≤2 weeks. Thirteen out of the 344 (4%) episodes experienced a recurrence of bacteremia within 120 days; in all of these subsequent episodes, cardiac imaging was performed, IE was ruled out, and a definitive infectious source was identified. During the same period, 131 of 344 (38%) patients died.

Among the 828 episodes of enterococcal bacteremia, 116 (14%), 146 (18%), and 133 (16%) episodes were classified as definite IE by the 2015 Duke-ESC, 2023 Duke-ISCVID, and 2023 Duke-ESC clinical criteria, respectively. Supplementary Table 3 details the 52 episodes that were diagnosed as IE by the Endocarditis Team but were categorized as either possible or rejected IE by either the 2023 Duke-ISCVID or 2023 Duke-ESC clinical criteria. Conversely, Supplementary Table 4 presents 17 episodes not diagnosed as IE by the Endocarditis Team but labeled definite IE by ≥1 Duke criteria version. In all of these discordant cases, IE diagnosis was subsequently ruled out, and no recurrence of bacteremia with the same *Enterococcus* spp. was observed within 120 days.

Among the 173 episodes of IE, a greater proportion fulfilled the major microbiological criterion under the updated 2023 Duke-ISCVID (158 episodes; 91%) and 2023 Duke-ESC (147 episodes; 85%) criteria compared with the 2015 Duke-ESC version (137 episodes; 79%). However, this broader inclusion came at the cost of reduced specificity: A significantly higher number of episodes without IE (n = 655) also met the major microbiological criterion when assessed with the 2023 Duke-ISCVID (300 episodes; 46%) and 2023 Duke-ESC (203 episodes; 31%) criteria, compared with the 2015 Duke-ESC version (37 episodes; 8%) (Table 2). As a result, more non-IE episodes were reclassified as possible IE under the 2023 criteria—287 (44%) episodes for Duke-ISCVID and 201 (31%) episodes for Duke-ESC—compared with only 91 (14%) episodes under the 2015 Duke-ESC framework.

**Table 2.** Classifications Based on the Three Versions of the Duke Clinical Criteria Among 828 Episodes With Enterococcal Bacteremia

	No Infective Endocarditis (n = 655), No. (%)	Infective Endocarditis (n = 173), No. (%)
Duke major clinical criteria		
Major imaging criterion (2015 ESC)	0 (0)	129 (75)
Major imaging criterion (2023 ISCVID)	7 (1)	139 (80)
Major imaging criterion (2023 ESC)	3 (0.5)	138 (80)
Major surgery criterion (2023 ISCVID)	0 (0)	0 (0)
Major microbiological criterion (2015 ESC)	37 (6)	137 (79)
Major microbiological criterion (2023 ISCVID)	300 (46)	158 (91)
Major microbiological criterion (2023 ESC)	203 (31)	147 (85)
Duke minor clinical criteria		
Minor microbiological criterion (2015 ESC)	618 (94)	33 (19)
Minor microbiological criterion (2023 ISCVID)	123 (19)	8 (5)
Minor microbiological criterion (2023 ESC)	136 (21)	7 (4)
Minor predisposition criterion (2015 ESC)	56 (9)	92 (53)
Minor predisposition criterion (2023 ISCVID)	133 (20)	135 (78)
Minor predisposition criterion (2023 ESC)	110 (17)	119 (69)
Minor vascular criterion (2015 ESC)	22 (3)	65 (3)
Minor vascular criterion (2023 ISCVID)	22 (3)	65 (38)
Minor vascular criterion (2023 ESC)	31 (5)	75 (43)
Minor immunological criterion (all versions)	0 (0)	8 (5)
Minor fever criterion (all versions)	555 (85)	140 (81)
Classification according to 2015 Duke-ESC clinical criteria		
Rejected	564 (86)	12 (7)
Possible	91 (14)	45 (26)
Definite	0 (0)	116 (67)
Classification according to 2023 Duke-ISCVID clinical criteria		
Rejected	359 (55)	2 (1)
Possible	287 (44)	34 (20)
Definite	9 (1)	137 (79)
Classification according to 2023 Duke-ESC clinical criteria		
Rejected	449 (69)	4 (2)
Possible	201 (31)	41 (24)
Definite	5 (0.8)	128 (74)

Abbreviations: ESC, European Society of Cardiology; ISCVID, International Society of Cardiovascular Infectious Diseases.

Table 3 summarizes the diagnostic performance of the 3 Duke clinical criteria versions using 2 analytical approaches. In both analyses, the sensitivity was 67% (95% CI, 59%–74%) for the 2015 Duke-ESC criteria, 79% (95% CI, 72%–85%) for the 2023 Duke-ISCVID criteria, and 74% (95% CI, 67%–80%) for the 2023 Duke-ESC criteria. Specificity in the first analysis was 100% (95% CI, 99%–100%) for the 2015 Duke-ESC, 98% (95% CI, 97%–99%) for the 2023 Duke-ISCVID, and 99% (95% CI, 98%–100%) for the 2023 Duke-ESC criteria. However, in the second analysis, specificity declined to 86% (95% CI, 83%–89%) for the 2015 Duke-ESC, 55% (95% CI, 51%–59%) for the 2023 Duke-ISCVID, and 69% (95% CI, 65%–72%) for the 2023 Duke-ESC criteria. Table 4 shows the comparative performance of the 3 Duke criteria versions alongside risk stratification scores specifically within the 650 episodes of enterococcal bacteremia from CHUV's bacteremia cohort.

**Table 3.** Performance of the Three Versions of the Duke Clinical Criteria for the Diagnosis of Infective Endocarditis Among 828 Episodes With Enterococcal Bacteremia, With the Reference Standard Being the Diagnosis of the Endocarditis Team

	Sensitivity (95% CI), %	Specificity (95% CI), %	PPV (95% CI), %	NPV (95% CI), %	Accuracy (95% CI), %
First analysis^a^					
2015 Duke-ESC	67 (59–74)	100 (99–100)	100 (97–100)	93 (90–93)	93 (91–95)
2023 Duke-ISCVID	79 (72–85)	98 (97–99)	90 (82–95)	95 (93–96)	94 (92–96)
2023 Duke-ESC	74 (67–80)	99 (98–100)	96 (91–98)	94 (92–95)	94 (92–95)
Second analysis^b^					
2015 Duke-ESC	67 (59–74)	86 (83–89)	56 (51–61)	91 (89–92)	82 (79–85)
2023 Duke-ISCVID	79 (72–85)	55 (51–59)	32 (29–34)	91 (88–93)	60 (56–63)
2023 Duke-ESC	74 (67–80)	69 (65–72)	38 (35–42)	91 (89–93)	70 (66–73)

Abbreviations: ESC, European Society of Cardiology; IE, infective endocarditis; ISCVID, International Society of Cardiovascular Infectious Diseases; NPV, negative predictive value; PPV, positive predictive value.

^a^Patients without IE (per reference standard) classified as possible IE (per Duke clinical criteria) were considered true negatives.

^b^Patients without IE (per reference standard) classified as possible IE (per Duke clinical criteria) were considered false positives.

**Table 4.** Performance of the Three Versions of the Duke Clinical Criteria for the Diagnosis of Infective Endocarditis Among 650 Episodes From the Bacteremia Cohort of the Lausanne University Hospital, With the Reference Standard Being the Diagnosis of the Endocarditis Team

	Sensitivity (95% CI), %	Specificity (95% CI), %	PPV (95% CI), %	NPV (95% CI), %	Accuracy (95% CI), %
First analysis^a^					
2015 Duke-ESC	62 (46–76)	100 (99–100)	100 (95–100)	97 (96–98)	97 (96–98)
2023 Duke-ISCVID	79 (64–89)	99 (97–99)	82 (70–90)	98 (97–99)	97 (96–98)
2023 Duke-ESC	72 (57–84)	99 (98–100)	89 (76–96)	98 (97–99)	97 (96–98)
Second analysis^b^					
2015 Duke-ESC	62 (46–76)	88 (85–91)	29 (23–36)	97 (95–98)	86 (83–89)
2023 Duke-ISCVID	79 (64–89)	57 (52–61)	12 (11–14)	97 (95–99)	58 (54–62)
2023 Duke-ESC	72 (57–84)	71 (67–75)	16 (14–19)	97 (95–98)	71 (67–75)

Abbreviations: ESC, European Society of Cardiology; IE, infective endocarditis; ISCVID, International Society of Cardiovascular Infectious Diseases; NPV, negative predictive value; PPV, positive predictive value.

^a^Patients without IE (per reference standard) classified as possible IE (per Duke clinical criteria) were considered true negatives.

^b^Patients without IE (per reference standard) classified as possible IE (per Duke clinical criteria) were considered false positives.

## DISCUSSION

In this combined cohort, the updated 2023 Duke-ISCVID and 2023 Duke-ESC clinical criteria offered improved sensitivity for diagnosing IE compared with the 2015 Duke-ESC criteria, albeit with a trade-off in specificity.

The prevalence of IE (CHUV's bacteremia cohort) was 7%, consistent with findings from earlier studies [23, 24]. Consistent with existing literature, IE was more frequently associated with *E. faecalis* bacteremia than with *E. faecium* and other enterococcal species [1, 23]. This disparity is likely driven by 2 key factors: (1) *E. faecalis* infections are more often community or health care associated rather than nosocomial [1, 23], and (2) *E. faecalis* expresses a wider repertoire of virulence factors, promoting enhanced epithelial adhesion and biofilm formation [25, 26]. Reflecting these characteristics, the 2023 ESC guidelines recommend routine echocardiographic evaluation in all cases of *E. faecalis* bacteremia [8]. The prevalence of IE among patients with *E. faecalis* bacteremia in our study was consistent with existing literature, which reports rates ranging from 11% to 17% [23, 27–29], with the notable exception of the study by Dahl et al., which found a higher prevalence of 26% [11]. This discrepancy is likely attributable to the systematic use of TTE in the study population [11]. By contrast, not all patients in our cohort, or in similar studies [23, 27–29], underwent cardiac imaging. Importantly, in our study, none of the patients who did not undergo cardiac imaging developed IE during a 120-day follow-up, despite the fact that nearly all (99%) received short-course antimicrobial therapy of ≤2 weeks—an approach that would typically be inadequate tor treating undiagnosed IE.

In previous studies, the 2015 Duke-ESC criteria showed improved sensitivity over the 2000 modified Duke criteria, primarily due to the inclusion of positive cardiac CT findings and abnormal metabolic activity on ^18^F-FDG PET/CT in prosthetic valves as part of the major imaging criterion [14, 15, 30]. In our study, both 2023 versions of the Duke criteria demonstrated improved sensitivity for diagnosing IE in patients with enterococcal bacteremia compared with the 2015 Duke-ESC criteria. This improvement was largely driven by revisions to the major microbiological criterion. However, the enhanced sensitivity came at the cost of a substantial increase in the number of non-IE cases being classified as possible IE, 44% with the 2023 Duke-ISCVID and 30% with the 2023 Duke-ESC criteria, vs only 5% with the 2015 version. These findings align with previous studies assessing the performance of the updated criteria across different clinical settings [12–19].

A major challenge in evaluating the diagnostic performance of the Duke criteria lies in the interpretation of “possible IE” cases. This category represents a diagnostically indeterminate group that often does not lead to treatment for IE but still requires further clinical evaluation. In our study, nearly one-third of episodes were classified as possible IE, underscoring the limited discriminative power of the criteria in their current form. While such cases are often managed conservatively in routine clinical practice, we considered them in 2 distinct ways in our analyses. In the first analysis, possible IE cases that were ultimately adjudicated as not IE were treated as true negatives, aligning with both clinical practice and prior studies [12–19]. In the second analysis, we classified these cases as false positives, as we believe this better captures the limitations in specificity and highlights how certain components of the revised criteria may lead to overdiagnosis. Accordingly, patients adjudicated as not having IE by the reference standard but labeled possible IE were reclassified as false positives. This adjustment led to a notable decrease in specificity across all versions of the Duke criteria. This dual approach underscores the interpretative nature of the “possible IE” category and highlights the need for future refinements to reduce diagnostic ambiguity. The reduction in specificity observed with the 2023 versions of the criteria was primarily driven by changes to the major microbiological criterion. Both the 2023 Duke-ISCVID and 2023 Duke-ESC criteria broaden the list of typical IE pathogens to include *E. faecalis*, even in nosocomial infections with an identifiable source [8, 9]. Additionally, the 2023 Duke-ISCVID criteria further extends this criterion by considering any enterococcal species to be typical pathogens when ≥3 blood culture sets are positive regardless of the clinical context [9].

This study has several limitations. First, its retrospective design and restriction to 2 tertiary care centers may limit generalizability. However, to our knowledge, this is the first study to specifically evaluate the 2023 revisions of the Duke criteria in a cohort of patients with enterococcal bacteremia. Second, the inclusion of patients from CHUV's cohort of suspected IE and the USZ IE registry resulted in an overrepresentation of endocarditis cases, which may have inflated sensitivity estimates. To mitigate this potential selection bias, we conducted a separate analysis using only CHUV's unselected bacteremia cohort, which more closely reflects routine clinical populations. Third, cardiac imaging was not performed in 42% of episodes. However, nearly all of these patients received short-course antibiotic treatment (≤2 weeks), and none developed enterococcal IE within 120 days of follow-up, supporting the assumption that IE was unlikely in these cases. Finally, the reference standard was based on adjudication by Endocarditis Teams and expert clinicians, which may have introduced classification bias. To address this, we transparently reported all discordant cases in which the adjudicated diagnosis differed from the classification assigned by the Duke criteria.

In conclusion, while the 2023 Duke criteria improved sensitivity for diagnosing IE in enterococcal bacteremia—mainly through the inclusion of *E. faecalis* as a typical pathogen—this gain came at the cost of reduced specificity. These findings highlight the need for further refinement before widespread clinical adoption. Future versions of the Duke criteria should undergo systematic evaluation of each component's diagnostic performance and consider stratifying definitions based on clinical context and patient population to better balance sensitivity and specificity in real-world practice.

## Supplementary Material

ofaf408_Supplementary_Data

## Supplementary Data

Supplementary materials are available at *Open Forum Infectious Diseases* online. Consisting of data provided by the authors to benefit the reader, the posted materials are not copyedited and are the sole responsibility of the authors, so questions or comments should be addressed to the corresponding author.
