# Supplementary material for: Evaluation of Different Versions of the Duke Criteria for the Diagnosis of Infective Endocarditis Among Patients With Enterococcal Bacteremia; a Multicenter Study
Source: Open Forum Infect Dis. 2025 Jul 4;12(7):ofaf408. doi: 10.1093/ofid/ofaf408 (PMC12287628; doi:10.1093/ofid/ofaf408)
Supplement: ofaf408_Supplementary_Data [file ofaf408_supplementary_data.pdf]

**Supplementary Table 1.** Diagnoses of episodes with recurrent bacteremia caused by the same enterococcal species within 120 days of the initial episode

| | Species | Diagnosis of the initial episode | Diagnosis of the subsequent episode |
|---|---|---|---|
| 1 | *E. faecalis* | Infective endocarditis | Infective endocarditis |
| 2 | *E. faecalis* | Infective endocarditis | Infective endocarditis |
| 3 | *E. faecalis* | Infective endocarditis | Infective endocarditis |
| 4 | *E. faecalis* | Infective endocarditis | Infective endocarditis |
| 5 | *E. faecalis* | Infective endocarditis | Infective endocarditis |
| 6 | *E. faecalis* | Infective endocarditis | Infective endocarditis |
| 7 | *E. faecalis* | Infective endocarditis | Catheter-related |
| 8 | *E. faecalis* | Unknown origin | Infective endocarditis |
| 9 | *E. faecalis* | Unknown origin | Infective endocarditis |
| 10 | *E. faecalis* | Unknown origin | Unknown origin |
| 11 | *E. faecalis* | Urinary-tract | Infective endocarditis |
| 12 | *E. faecalis* | Urinary-tract | Spondylodiscitis |
| 13 | *E. faecalis* | Urinary-tract | Spondylodiscitis |
| 14 | *E. faecalis* | Urinary-tract | Urinary-tract |
| 15 | *E. faecalis* | Urinary-tract | Urinary-tract |
| 16 | *E. faecalis* | Urinary-tract | Catheter-related |
| 17 | *E. faecalis* | Catheter-related | Skin and soft tissue infection complicating chronic osteitis |

| | | | |
|---|---|---|---|
| 18 | *E. faecalis* | Catheter-related | Unknown origin |
| 19 | *E. faecalis* | Catheter-related | Catheter-related |
| 20 | *E. faecalis* | Cholangitis | Cholangitis |
| 21 | *E. faecalis* | Cholangitis | Cholangitis |
| 22 | *E. faecalis, E. faecium* | Cholangitis | Cholangitis |
| 23 | *E. faecium* | Cholangitis | Cholangitis |
| 24 | *E. faecium* | Cholangitis | Cholangitis |
| 25 | *E. faecium* | Cholangitis | Cholangitis |
| 26 | *E. faecium* | Cholangitis | Cholangitis |
| 27 | *E. faecium* | Cholangitis | Cholangitis |
| 28 | *E. faecium* | Cholangitis | Cholangitis |
| 29 | *E. faecium* | Cholangitis | Cholangitis |
| 30 | *E. faecium* | Cholangitis | Cholangitis |
| 31 | *E. faecium* | Cholangitis | Cholangitis |
| 32 | *E. faecium* | Cholangitis | Cholangitis |
| 33 | *E. faecium* | Cholangitis | Cholangitis |
| 34 | *E. faecium* | Cholangitis | Cholangitis |
| 35 | *E. faecium* | Cholangitis | Cholangitis |
| 36 | *E. faecium* | Catheter-related | Catheter-related |

| | | | |
|---|---|---|---|
| 37 | *E. faecium* | Catheter-related | Catheter-related |
| 38 | *E. faecium* | Catheter-related | Septic thrombosis of portal vein |
| 39 | *E. faecium* | Urinary-tract | Urinary-tract |

**Supplementary Table 2.** Prevalence of infective endocarditis of different enterococcal species among 650 episodes from the bacteremia cohort

| | **No infective endocarditis (n=603)** | **Infective endocarditis (n=47)** | **Prevalence of infective endocarditis** |
|---|---|---|---|
| *E, faecalis*, n (%) | 294 (49) | 41 (87) | 12% |
| *E. faecium, n (%)* | 289 (48) | 6 (13) | 2% |
| Other enterococcus sp, n (%) | 46 (8) | 1 (2) | 2% |

**Supplementary Table 3.** Episodes of infective endocarditis (Endocarditis Team or expert clinicians' classification) categorized as rejected or possible infective endocarditis by either the 2023 ISCVID or 2023 ESC Duke clinical criteria

| | **Microbiologic criterion** | **Imaging criterion** | **Predisposition** | **Fever** | **Vascular or immunologic phenomena** | **Other information** |
|---|---|---|---|---|---|---|
| 1 | *E. faecium* (3 bcs) | Valve vegetation, abnormal metabolic activity ($^{18}$F-FDG PET/CT) of TAVI | TAVI, CIED | Y | | |
| 2 | *E. faecium* (2 bcs) | Valve vegetation, CIED-lead vegetation | Prosthetic valve, CIED | Y | | Cardiac surgery; macroscopic signs of IE during surgery; valve culture positive for *E. faecium*; CIED extraction |
| 3 | *E. faecium* (2 bcs) | Valve vegetation | Prosthetic valve | Y | | |
| 4 | *E. faecium* (2 bcs) | Valve vegetation | Prosthetic valve | Y | | |
| 5 | *E. faecium* (2 bcs) | Abnormal metabolic activity ($^{18}$F-FDG PET/CT) of TAVI | TAVI | Y | | |

| | | | | | | |
|---|---|---|---|---|---|---|
| 6 | *E. faecium* (2 bcs) | Valve abscess | Prosthetic valve, prior IE, CIED | Y | | |
| 7 | *E. faecalis* (1 bcs) | Valve abscess | | Y | | Only one blood culture set was dawn before antimicrobial treatment initiation; cardiac surgery; macroscopic signs of IE during surgery; valve culture positive for *E. faecalis* |
| 8 | *E. faecalis* (1 bcs) | Valve vegetation | Moderate valve regurgitation | Y | | Only one blood culture set was dawn before antimicrobial treatment initiation; cardiac surgery; positive pathology |
| 9 | *E. faecalis* (2 bcs) | | Prosthetic valve, CIED | Y | | Community-acquired bacteremia of unknown origin |
| 10 | *E. faecalis* (2 bcs) | | Moderate valve regurgitation | Y | Janeway lesions | Community-acquired bacteremia of unknown origin |
| 11 | *E. faecalis* (2 bcs) | Significant new valvular regurgitation | Bicuspid aortic valve | Y | | Community-acquired bacteremia of unknown origin |
| 12 | *E. faecium* (4 bcs) | Valve vegetation | | N | Cerebral emboli | |
| 13 | *E. faecalis* (2 bcs) | Significant new valvular regurgitation | Prosthetic valve, prior IE, CIED | Y | | Community-acquired bacteremia of unknown origin |

| | | | | | | |
|---|---|---|---|---|---|---|
| 14 | *E. faecalis* (3 bcs) | Significant new valvular regurgitation | Prior IE | Y | | Community-acquired bacteremia of unknown origin |
| 15 | *E. faecium* (1 bcs) | Valve vegetation | Surgical valve repair (within 6 months); moderate valve regurgitation | Y | | Cardiac surgery; macroscopic signs of IE during surgery |
| 16 | *E. faecium* (2 bcs) | Significant new valvular regurgitation | TAVI, CIED | Y | | Community-acquired bacteremia of unknown origin |
| 17 | *E. faecalis* (2 bcs) | Significant new valvular regurgitation | | N | | Community-acquired bacteremia of unknown origin |
| 18 | *E. faecalis* (2 bcs) | Valve leaflet thickening | Prosthetic valve | Y | | Community-acquired bacteremia of unknown origin |
| 19 | *E. faecalis* (3 bcs) | | Prosthetic valve | Y | Spondylodiscitis | Community-acquired bacteremia |
| 20 | *E. faecium* (2 bcs) | Valve leaflet thickening | Prosthetic valve, CIED | Y | Splenic emboli | Community-acquired bacteremia of unknown origin; CIED extraction at day 36; CIED lead culture negative |

| | | | | | | |
|---|---|---|---|---|---|---|
| 21 | *E. faecalis* (3 bcs) | Valve leaflet thickening | | Y | Septic arthritis | Community-acquired bacteremia |
| 22 | *E. faecalis* (3 bcs) | Valve leaflet thickening | TAVI, CIED | Y | | Community-acquired bacteremia of unknown origin |
| 23 | *E. faecalis* (1 bcs) | Valve leaflet thickening; significant new valvular regurgitation | | Y | Septic arthritis | community-acquired bacteremia; only one blood culture set was dawn before antimicrobial treatment initiation |
| 24 | *E. faecium* (2 bcs) | Valve vegetation | Prosthetic valve | N | | Cardiac surgery; macroscopic signs of IE during surgery; positive pathology |
| 25 | *E. faecium* (2 bcs) | Valve vegetation, CIED-lead vegetation | Prosthetic valve, CIED | Y | | Cardiac surgery; macroscopic signs of IE during surgery; positive pathology; CIED extraction |
| 26 | *E. faecium* (2 bcs) | Valve vegetation | TAVI, CIED | Y | | |
| 27 | *E. avium* (2 bcs) | Valve vegetation | | N | Splenic emboli | Cardiac surgery; macroscopic signs of IE during surgery |
| 28 | *E. faecalis* (3 bcs) | | Prosthetic valve, CIED | N | Splenic emboli | Community-acquired bacteremia of unknown origin; persistent bacteremia for at least 48h |

| | | | | | |
|---|---|---|---|---|---|
| 29 | *E. faecalis* (3 bcs) | | Moderate valve regurgitation | Y | Community-acquired bacteremia of unknown origin |
| 30 | *E. faecalis* (2 bcs) | | TAVI, prior IE | Y | Community-acquired bacteremia of unknown origin |
| 31 | *E. faecalis* (2 bcs) | | TAVI | Y | Community-acquired bacteremia of unknown origin |
| 32 | *E. faecalis* (3 bcs) | | Prosthetic valve, CIED | Y | Community-acquired bacteremia of unknown origin |
| 33 | *E. faecalis* (3 bcs) | Small valve mobile element | Prosthetic valve, CIED | Y | Community-acquired bacteremia of unknown origin |
| 34 | *E. faecalis* (3 bcs) | | TAVI, CIED | Y | Community-acquired bacteremia of unknown origin |
| 35 | *E. faecalis* (2 bcs) | | CIED | Y | Community-acquired bacteremia of unknown origin; CIED extraction at day 7; CIED lead culture negative |
| 36 | *E. faecalis* (1 bcs) | | Prosthetic valve | Y | Community-acquired bacteremia of unknown origin; Only one blood culture set was dawn before antimicrobial treatment initiation |
| 37 | *E. faecalis* (2 bcs) | | TAVI, prior IE | Y | Community-acquired bacteremia of unknown origin |
| 38 | *E. faecalis* (2 bcs) | Significant new valvular regurgitation | Prosthetic valve, prior IE | Y | Community-acquired bacteremia of unknown origin |
| 39 | *E. faecalis* (2 bcs) | | Prosthetic valve | Y | Community-acquired bacteremia of unknown origin |

| | | | | | | |
|---|---|---|---|---|---|---|
| 40 | *E. faecium* (1 bcs) | Valve vegetation, perforation, significant new valvular regurgitation | | N | Cerebral emboli, splenic emboli | Only one blood culture set was dawn before antimicrobial treatment initiation |
| 41 | *E. faecium* (2 bcs) | CIED-lead vegetation; abnormal metabolic activity ($^{18}$F-FDG PET/CT) of CIED-lead | CIED | N | | Community-acquired bacteremia of unknown origin |
| 42 | *E. faecalis* (2 bcs) | | | Y | | Community-acquired bacteremia of unknown origin |
| 43 | *E. faecalis* (2 bcs) | | CIED | Y | | Community-acquired bacteremia of unknown origin |
| 44 | *E. faecalis* (2 bcs) | | | N | Cerebral emboli, Janeway lesions, Roth spots | Community-acquired bacteremia of unknown origin |
| 45 | *E. faecalis* (3 bcs) | | Prosthetic valve, prior IE | Y | | Community-acquired bacteremia of unknown origin |

| | | | | | |
|---|---|---|---|---|---|
| 46 | *E. faecalis* (3 bcs) | | CIED | N | Community-acquired bacteremia of unknown origin; persistent bacteremia for at least 48h |
| 47 | *E. faecalis* (1 bcs) | | Moderate valve regurgitation | Y | Community-acquired bacteremia of unknown origin |
| 48 | *E. faecalis* (1 bcs) | | | Y | Community-acquired bacteremia of unknown origin; persistent bacteremia for at least 48h; on antibiotic treatment before blood cultures were drawn |
| 49 | *E. faecalis* (1 bcs) | | Prosthetic valve, CIED | Y | Community-acquired bacteremia of unknown origin; only one blood culture set was dawn before antimicrobial treatment initiation persistent bacteremia for at least 48h; only one blood culture set was dawn before antimicrobial treatment initiation |
| 50 | *E.  faecium* (2 bcs) | Abnormal metabolic activity ($^{18}$F-FDG PET/CT) of native valve | CIED | N | |
| 51 | *E. faecalis* (2 bcs) | | Moderate valve regurgitation, LVAD | Y | Community-acquired bacteremia of unknown origin |

| | | | | | |
|---|---|---|---|---|---|
| 52 | *E. faecalis* (1 bcs) | Valve leaflet thickening | Y | Septic arthritis | Community-acquired bacteremia; on antibiotic treatment before blood cultures were drawn |

$^{18}$F-FDG PET/CT: $^{18}$F-Fluorodeoxyglucose Positron Emission Tomography/Computed Tomography; bcs: blood culture set; CIED: cardiac implantable electronic device; ESC: European Society of Cardiology; IE: infective endocarditis; ISCVID: International Society of Cardiovascular Infectious Diseases; IVDU: intravenous drug use; LVAD: left ventricular assist device; N: no; TAVI: transcatheter aortic valve implantation; TEE: transesophageal echocardiography; Y: yes

**Supplementary Table 4.** Episodes without infective endocarditis (Endocarditis Team or expert clinicians' classification) categorized as definite infective endocarditis by any version of the Duke clinical criteria

| | **Microbiologic criterion** | **Imaging criterion** | **Predisposition** | **Fever** | **Vascular or immunologic phenomena** | **Other information** |
|---|---|---|---|---|---|---|
| 1 | *E. faecalis* (2 bcs) | | Prosthetic valve | Y | Janeway lesions | Septic shock due to urinary-tract infection; petechias mistaken for Janeway lesions; TEE negative for IE; antibiotic treatment for less than 14 days; no episode of bacteremia/IE in the subsequent 120 days |
| 2 | *E. faecalis* (2 bcs) | Significant new valvular regurgitation [a] | Moderate valvular regurgitation[a] | Y | | Nosocomial bacteremia; abdominal infection; TEE negative for IE; antibiotic treatment for less than 14 days; no episode of bacteremia/IE in the subsequent 120 days |
| 3 | *E. faecalis* (2 bcs)<br>E. coli (2 bcs) | Significant new valvular regurgitation[a] | Moderate valvular regurgitation[a] | Y | | Nosocomial bacteremia; abdominal infection; TEE negative for IE; antibiotic treatment for less than 14 days; no episode of bacteremia/IE in the subsequent 120 days |
| 4 | *E. faecalis* (2 bcs) | Significant new valvular regurgitation | | Y | | Nosocomial bacteremia; catheter-related bacteremia; TEE negative for IE; antibiotic treatment for less than 14 days; no episode of bacteremia/IE in the subsequent 42 days |

| | | | | | | |
|---|---|---|---|---|---|---|
| 5 | *E. faecalis* (2 bcs)<br>E. coli (2 bcs) | | Moderate valvular regurgitation | Y | Cerebral emboli | Nosocomial bacteremia; abdominal infection; TEE negative for IE; ischemic stroke at day 11 due to atrial fibrillation |
| 6 | *E. faecalis* (2 bcs) | Significant new valvular regurgitation | | Y | | Nosocomial bacteremia; urinary-tract infection |
| 7 | *E. faecalis* (2 bcs) | Significant new valvular regurgitation | | Y | | Nosocomial bacteremia; urinary-tract infection; antibiotic treatment for less than 14 days; no episode of bacteremia/IE in the subsequent 120 days |
| 8 | *E. faecalis* (2 bcs)<br>*E. coli* (2 bcs) | Significant new valvular regurgitation | | Y | | Urinary-tract infection; antibiotic treatment for less than 14 days; no episode of bacteremia/IE in the subsequent 120 days |
| 9 | *E. faecium* (3 bcs) | Significant new valvular regurgitation | | Y | | Nosocomial bacteremia; abdominal infection; antibiotic treatment for less than 14 days; no episode of bacteremia/IE in the subsequent 120 days |
| 10 | *E. raffinosus* (2 bcs)<br>*K. pneumoniae* (2 bcs) | Significant new valvular regurgitation | | N | | Abdominal infection; antibiotic treatment for less than 14 days; no episode of bacteremia/IE in the subsequent 120 days |

| | | | | | | |
|---|---|---|---|---|---|---|
| 11 | *E. faecalis* (3 bcs) | Significant new valvular regurgitation | Prior IE | N | | Catheter-related bacteremia; TEE and $^{18}$F-FDG PET/CT negative for IE; antibiotic treatment for less than 14 days; no episode of bacteremia/IE in the subsequent 120 days |
| 12 | *E. faecalis* (3 bcs) | Significant new valvular regurgitation | CIED | N | | Bacteremia of unknown origin; TEE and $^{18}$F-FDG PET/CT negative for IE; antibiotic treatment for less than 14 days; no episode of bacteremia/IE in the subsequent 22 days |
| 13 | *E. faecalis* (2 bcs) | Significant new valvular regurgitation | CIED | Y | | Nosocomial bacteremia; TEE and $^{18}$F-FDG PET/CT negative for IE; antibiotic treatment for less than 14 days; no episode of bacteremia/IE in the subsequent 120 days |
| 14 | *E. faecium* (3 bcs) | | CIED | Y | Pulmonary emboli | Catheter-related infection with septic deep vein thrombosis; $^{18}$F-FDG PET/CT negative for IE; antibiotic treatment for less than 14 days; no episode of bacteremia/IE in the subsequent 29 days |
| 15 | *E. faecalis* (3 bcs) *K. pneumoniae* (3 bcs) | | Prosthetic valve | Y | Spondylodiscitis | TEE and $^{18}$F-FDG PET/CT negative for IE; no episode of bacteremia/IE in the subsequent 120 days |
| 16 | *E. faecalis* (3 bcs) | Valve leaflet thickening | Prosthetic valve | Y | | Nosocomial bacteremia; urinary-tract infection; TEE negative for IE; antibiotic treatment for less than 14 days; no episode of bacteremia/IE in the subsequent 120 days |

| | | | | |
|---|---|---|---|---|
| 17 | *E. faecalis* (3 bcs) | Valve leaflet thickening | N | Catheter-related bacteremia; TEE negative for IE; antibiotic treatment for less than 14 days; no episode of bacteremia/IE in the subsequent 120 days |

$^{18}$F-FDG PET/CT: $^{18}$F-Fluorodeoxyglucose Positron Emission Tomography/Computed Tomography; bcs: blood culture set; CIED: cardiac implantable electronic device; IE: infective endocarditis; N: no; TEE: transesophageal echocardiography; Y: yes

[a]the significant new valvular regurgitation (imaging criterion) appeared in a different valve from the moderate valvular regurgitation previously observed (predisposition criterion)
